# Thyrotoxic Periodic Paralysis and Cardiomyopathy in a Patient with Graves’ Disease

**DOI:** 10.7759/cureus.2837

**Published:** 2018-06-19

**Authors:** Anna A Abbasi, Prarthna Chandar, Shyam Shankar, Sushilkumar S Gupta, Yizhak Kupfer

**Affiliations:** 1 Internal Medicine, Maimonides Medical Center, Brooklyn, USA; 2 Critical Care, Maimonides Medical Center, Brooklyn, USA

**Keywords:** thyrotoxicosis, thyrotoxic cardiomyopathy, thyrotoxic periodic paralysis, icu management

## Abstract

Thyrotoxic periodic paralysis (TPP) and cardiomyopathy are two established complications of thyrotoxicosis. Emergent management is essential as TPP and cardiac events secondary to thyrotoxic cardiomyopathy can be fatal. We report a unique case of a patient with Graves’ disease presenting with symptoms secondary to both these complications. A 34-year-old Hispanic male, diagnosed with Graves’ disease, non-compliant with his medications, presented to the emergency room (ER) with complaints of generalized weakness, palpitations, chest pain and multiple episodes of nausea and vomiting for one day. On presentation, the patient was tachycardiac, had a systolic flow murmur and decreased motor strength in all extremities. Blood work showed a potassium of 1.8 millimoles per liter, cardiac troponin of 0.04 nanograms per milliliter and a thyroid panel consistent with hyperthyroidism. Electrocardiogram showed atrial flutter. In the ER, Propranolol, Propylthiouracil and Hydrocortisone were administered to prevent thyroid storm. Potassium was repleted, and the patient developed rebound hyperkalemia. He was given calcium gluconate, insulin, sodium polystyrene and admitted to the medical intensive care unit (MICU) for further management. Echocardiogram revealed severely decreased left ventricular systolic function and an ejection fraction of 26-30%. He was diagnosed with cardiomyopathy secondary to thyrotoxicosis. He was stabilized with Methimazole, Propranolol, Lisinopril and discharged on day nine with these medications and an outpatient follow-up appointment. Thyrotoxicosis can be life-threatening. This case shows a unique instance where a Hispanic patient presented with two complications of this phenomena. The pathogenesis of TPP involves increased responsiveness of the beta-adrenergic receptors, which leads to increased activity of the Sodium/Potassium (Na^+^/K^+^) ATPase pump and a transcellular shift of potassium into cells. The condition can resolve acutely with the administration of potassium. It is important to monitor the rate of potassium replacement as rebound hyperkalemia can occur, as this case demonstrates. Propranolol is an integral part of treatment as it is a beta-adrenergic receptor blocker and blocks the peripheral conversion of thyroxine (T4) to triiodothyronine (T3) in high doses. Thyrotoxic cardiomyopathy is one of the many cardiac complications that can be precipitated by Graves’ disease. One probable cause is the chronic tachycardia that patients with hyperthyroidism develop. Treatment entails managing the hyperthyroidism by starting the patient on beta blockers and anti-thyroid drugs or radioactive iodine uptake. Diuretics can be started to manage patients with heart failure. It is important to identify and treat the condition immediately to prevent grave complications.

## Introduction

Thyrotoxic periodic paralysis (TPP) and cardiomyopathy are two established complications of thyrotoxicosis [1-3]. A patient suffering from thyrotoxicosis can present acutely with these complications. Presenting symptoms can include, but are not limited to chest pain, palpitations, dyspnea, tachycardia and generalized weakness [2, 4]. Emergent management is essential as TPP and cardiac events secondary to thyrotoxic cardiomyopathy can be life-threatening. We report a case of a patient with Graves’ disease presenting with symptoms secondary to these complications.

## Case presentation

A 34-year-old Hispanic male, diagnosed with Graves’ Disease three years prior to presentation, non-compliant with his medications, presented to the emergency room (ER) with complaints of generalized weakness, palpitations, chest pain and multiple episodes of nausea and vomiting. The patient had been in his usual state of health till a day before admission. Vitals showed his blood pressure to be 137/83 mmHg and heart rate to be 119 beats per minute. Physical exam was significant for proptosis, a systolic flow murmur and upper and lower extremity weakness graded with a three out of five on the strength scale. Blood work showed a potassium of 1.8 millimoles per liter (mmol/l), thyroid stimulating hormone (TSH) 0.02 micro international units/milliliters (mcIU/ml), Free Triiodothyronine (T3) 25.14 picograms/milliliters (pg/ml) and Free Thyroxine (T4) 5.23 nanograms/deciliter (ng/dl). Cardiac troponin was 0.04 nanograms/milliliter (ng/ml). Electrocardiogram showed the patient to be in atrial flutter. In the ER, Propranolol was administered, along with Propylthiouracil and Hydrocortisone to prevent thyroid storm. Morphine was given to manage the pain and Ondansetron for the nausea and vomiting. A central line was placed through the Internal Jugular vein for rapid Potassium repletion. He developed rebound hyperkalemia with a potassium as high as 6.9 mmol/l. The patient was given calcium gluconate, insulin and sodium polystyrene. He was stabilized and admitted to the medical intensive care unit (MICU) for further management. In the MICU, the patient was switched from Propylthiouracil to Methimazole. Hydrocortisone was continued. He had an elevation in his cardiac troponin to 1.52 ng/ml, however, it trended down after the patient was hemodynamically stabilized. Echocardiogram revealed mild to moderately dilated left ventricle, mild to moderately dilated left atrium, severely decreased left ventricular systolic function and an ejection fraction of 26-30%. The patient was diagnosed with cardiomyopathy secondary to thyrotoxicosis. Management was continued with anti-thyroid drugs and Propranolol. On day two, the patient improved clinically, his weakness abated, he did not complain of any chest pain and he did not have any episodes of nausea and vomiting. Physical exam showed an increase in the upper and lower extremity muscle strength to five out of five. The patient's potassium was down to 4.2 mmol/l and thyroid function tests were repeated which showed the TSH, Free T3 and Free T4 trending down. The patient was downgraded to the regular medicine floor and discharged on day nine with Methimazole, Propranolol and Lisinopril, with an outpatient follow-up appointment.

## Discussion

TPP is a more common phenomenon in the Asian population [5], however, it has been reported in Hispanic males. It presents more often in men than women [5]. About 0.2% of the population affected by hyperthyroidism develops TPP [6]. Patients can present with generalized weakness (that affects the lower extremities more than the upper extremities), hyporeflexia and/or areflexia [2]. The pathogenesis involves increased responsiveness of the beta-adrenergic receptors, which in turn leads to increased activity of the Sodium/Potassium (Na^+^/K^+^) ATPase pump and a transcellular shift of potassium into cells [7]. Genetic mutations in the encoding of Kir2.6, a potassium channel in skeletal muscle that regulates membrane potential stability, make certain patients with Graves’ disease more susceptible to developing TPP [8]. The condition can resolve acutely with the administration of potassium, however, it is important to monitor the rate of potassium replacement as rebound hyperkalemia can occur in these patients, as this case demonstrates [5]. Rebound hyperkalemia develops when, upon the resolution of TPP the amount of potassium entering the extracellular fluid exceeds the renal potassium being excreted. During an episode of TPP, the overall stores of potassium in the body are not lowered [5]. Potassium is temporarily stored in cells due to the increased activity of the beta-adrenergic receptors. Propranolol is the other integral part of TPP treatment as it is a beta-adrenergic receptor blocker [5]. Administration of propranolol slows the activity of these receptors down, which along with potassium repletion can increase the serum potassium levels. In high doses propranolol blocks the conversion of T4 to T3 [9]. Thyrotoxic cardiomyopathy is one of the many cardiac complications that can be precipitated by Graves’ disease [3]. The mechanism of cardiomyopathy is not well understood, however, one probable cause is the chronic tachycardia that patients with hyperthyroidism develop [10]. Some other cardiac manifestations of hyperthyroidism include atrial fibrillation, congestive heart failure and pulmonary hypertension [3, 11]. The mortality from cardiogenic shock secondary to thyrotoxicosis is about 30% [12]. In most cases, the cardiovascular manifestations can be reversed by treating the hyperthyroidism [13]. Treatment entails starting the patient on beta blockers and anti-thyroid drugs or radioactive iodine uptake. Diuretics can be started to manage patients with heart failure [13]. Our patient was treated with and responded well to all these medications.

## Conclusions

Thyrotoxicosis can cause grave complications. Our patient presented with two separate, life-threatening complications of thyrotoxicosis secondary to Graves’ disease. It is important to identify and treat the condition immediately to reduce morbidity and prevent mortality from the life-threatening complications that can occur.

## References

[1] Allencherril J, Birnbaum I (2015). Heart failure in thyrotoxic cardiomopathy: extracorporeal membrane oxygenation treatment for Graves’ disease. J Extra Corpor Technol.

[2] Pompeo A, Nepa A, Maddestra M, Feliziani V, Genovesi N (2007). Thyrotoxic hypokalemic periodic paralysis: an overlooked pathology in western countries. Eur J Intern Med.

[3] Tsymbaliuk I, Unukovych D, Shvets N, Dinets A (2015). Cardiovascular complications secondary to Graves’ disease: a prospective study from Ukraine. Plos One.

[4] Osman F, Franklyn JA, Holder RL, Sheppard MC, Gammage MD (2007). Cardiovascular manifestations of hyperthyroidism before and after antithyroid therapy: a matched case-control study. J Am Coll Cardiol.

[5] Manoukian MA, Foote JA, Crapo LM (1999). Clinical and metabolic features of thyrotoxic periodic paralysis in 24 episodes. Arch Intern Med.

[6] Meseeha M, Parsamehr B, Kissell K, Attiac M (2017). Thyrotoxic periodic paralysis: a case study and review of the literature. J Community Hosp Intern Med Perspect.

[7] Rhee EP, Scott JA, Dighe AS (2012). Case 4-2012 — a 37-year-old man with muscle pain, weakness, and weight loss. N Engl J Med.

[8] Tella SH, Kommalapati A (2015). Thyrotoxic periodic paralysis: an underdiagnosed and under-recognized condition. Cureus.

[9] Wiersinga WM, Touber JL (1977). The influence of beta-adrenoceptor blocking agents on plasma thyroxine and triiodothyronine. J Clin Endocrinol Metab.

[10] Yu YH, Bilezikian JP (2000). Tachycardia-induced cardiomyopathy secondary to thyrotoxicosis: a young man with previously unrecognized Graves' disease. Thyroid.

[11] Lozano HF, Sharma CN (2004). Reversible pulmonary hypertension, tricuspid regurgitation and right-sided heart failure associated with hyperthyroidism: case report and review of the literature. Cardiol Rev.

[12] Nayak B, Burman K (2006). Thyrotoxicosis and thyroid storm. Endocrinol Metab Clin North Am.

[13] Klein I, Danzi S (2016). Thyroid disease and the heart. Curr Probl Cardiol.

